# Gut Microbiota and Metabolic Modulation by Slow-Release Protein Substitutes in Phenylketonuria: Findings from the PREMP Study

**DOI:** 10.3390/nu17243829

**Published:** 2025-12-07

**Authors:** Martina Tosi, Matteo Domenico Marsiglia, Emerenziana Ottaviano, Sara Parolisi, Juri Zuvadelli, Silvia Ancona, Camilla Ceccarani, Maria Teresa Carbone, Graziella Cefalo, Elisa Borghi, Elvira Verduci

**Affiliations:** 1 Department of Health Sciences, University of Milan, 20142 Milan, Italy; 2 Department of Pediatrics, Vittore Buzzi Children’s Hospital, 20154 Milan, Italy; 3 UOSD Metabolic Diseases, AORN Santobono-Pausilipon, 80122 Naples, Italy; s.parolisi@santobonopausilipon.it (S.P.);; 4 Clinical Department of Pediatrics, San Paolo Hospital, ASST Santi Paolo e Carlo, 20142 Milan, Italy; 5 Institute of Biomedical Technologies, National Research Council, 20054 Segrate, Italy

**Keywords:** Phenylketonuria, protein substitutes, slow-release amino acids, gut-microbiota, short chain fatty acids, insulin, glucose

## Abstract

**Background/Objectives**: Phenylketonuria (PKU) is an inherited metabolic disorder requiring early and lifelong dietary management through a low-phenylalanine (Phe) diet supplemented with Phe-free protein substitutes (PS). Recently developed slow-release PS formulations aim to mimic natural protein absorption, enhancing metabolic stability and tolerability. The PREMP study (effect of Protein RElease on the Microbiota composition and function in Phenylketonuric subjects) assessed the effects of a slow-release, Phe-free PS on gut microbiota composition and metabolic parameters in patients with PKU. **Methods**: Patients aged ≥6 years with PKU were enrolled from two Italian centers (Milan and Naples). Participants replaced ≥50% of their usual protein equivalent (P.Eq.) intake from Phe-free PS with a slow-release PS for 4 months. Clinical, biochemical, and nutritional assessments were performed at baseline and post-intervention. Gut microbiota composition was analyzed by 16S rRNA gene sequencing, and fecal fatty acids were quantified by gas chromatography–mass spectrometry. **Results**: Thirteen patients (median age 17 years) completed the intervention, replacing on average 78% of their usual P.Eq. intake with the slow-release formulation. Plasma phenylalanine and tyrosine levels remained stable, while fasting insulin (*p* = 0.0185) and HOMA-IR (*p* = 0.0099) significantly decreased, indicating improved insulin sensitivity. Anthropometric and dietary parameters showed no significant changes. Gut microbiota diversity remained stable, with modest increases in microbial richness and beneficial genera such as *Bacteroides*, *Bifidobacterium*, and *Gemmiger*, while *Hafnia, Anaerostipes* and *Romboutsia* decreased. Fecal butyrate and other fatty acids showed slight, non-significant increases. **Conclusions**: The slow-release PS was safe, well-tolerated, and improved insulin sensitivity without affecting amino acid or nutritional status. Microbial changes suggest potential benefits for gut health, warranting confirmation in larger, long-term studies.

## 1. Introduction

Phenylketonuria (PKU), OMIM: 261600, an autosomal recessive inherited metabolic disorder caused by mutations of Phenylalanine Hydroxylase enzyme (PAH), which converts phenylalanine (Phe) into tyrosine (Tyr), is characterized by high blood Phe concentrations that cause neurodevelopmental damage if left untreated [1]. The cornerstone of PKU treatment is nutritional intervention, consisting of restricted Phe intake through a restricted natural protein intake [2]. The European Guidelines for PKU management [1] indicate that a “low Phe diet” should be started as soon as possible, ideally within 10 days of life to prevent brain damage. Together with natural protein intake restriction, the integration with Phe-free protein substitutes (PS) and the consumption of low-protein foods represent the basis of dietary treatment in order to maintain Phe concentrations within safe ranges.

Thanks to advances in food technology, new PS have been developed that can slow the release of contained free amino acids (L-AAs), more closely mimicking the physiological absorption of intact natural proteins. These formulations aim to enhance protein metabolism while minimizing fluctuations in plasma amino acid concentrations [3]. The first slow-release PS, consisting of amino acids in alginate-based and coated micro tablets, was designed in 2002 [4]. In 2018, a novel prolonged-release PS containing L-AAs, coated with ethyl cellulose and alginate, was formulated. Moreover, the matrix and coating of these formulations enhance palatability by improving taste and reducing odor, thereby favoring consumption of the PS [4,5], and may also contribute to reducing gastrointestinal symptoms [5]. In children with PKU, a single prospective observational study, which replaced 20–30% of the standard protein supplement intake with a prolonged-release formulation, reported improved gastrointestinal tolerability [5]. Importantly, free L-AAs bypass the digestion phase, resulting in a different absorption profile compared to that of intact proteins, characterized by a faster peak and subsequent decrease in plasma [6]. Furthermore, the accelerated absorption of free L-AAs promotes higher rates of oxidation, protein breakdown, and urinary nitrogen loss [3,7]. The aim of slow- or prolonged-release PS is to provide a similar absorption profile to natural proteins, along with a better palatability to improve dietary adherence of patients [8]. In healthy adults, the administration of prolonged-release PS demonstrated to reduce the plasma peak of essential AAs (EAAs) while maintaining sustained overall bioavailability. In addition, studies have reported lower blood urea nitrogen (BUN) and urea excretion, a reduced insulin peak, and a less pronounced decline in blood glucose levels, which may contribute to a more physiological satiety response [9]. BUN is a parameter of AA oxidation and reflects the body’s capacity to retain dietary nitrogen derived from AAs [10]. In acute conditions, BUN can be used as a marker of AA oxidation indicating when anabolic pathways become saturated and supplemented AAs are no longer incorporated into protein synthesis [7,11], but rather catabolized and used for energy. In humans it has been demonstrated that proteins with a faster absorption kinetic determine greater insulin responses than proteins with a slower absorption rate. As a consequence, blood glucose levels are more strongly lowered with fast absorbed proteins [12]. Slowly absorbed proteins therefore moderate blood glucose fluctuations and support metabolic stability by steadying insulin release.

Several clinical studies have examined the gut microbiota composition in patients with PKU adhering to diet, consistently showing reduced microbial diversity along with significant taxonomic and functional changes [13]. To date, no data are available on the impact of slow-release PS on the composition and function of the intestinal microbiota in children with PKU. Preliminary findings [14,15] suggest that a low-Phe diet, characterized by a higher carbohydrate intake, results in a different quality of substrates for microbial fermentation, leading to a reduction in microbial richness at gut level. In particular, a decrease in two key butyrate-producing genera, *F. prausnitzii* and *Roseburia* spp., as well as a reduction in fecal butyrate, has been observed. In a recently published preclinical study in Caco-2 cells, slow-release PS showed good anti-oxidative and anti-inflammatory activity [16].

The aim of the PREMP study (effect of Protein RElease on the Microbiota composition and function in Phenylketonuric subjects) is to evaluate the potential effects of a slow-release, Phe-free PS (Afenil Micro 3H, Piam Farmaceutici S.p.a., Genoa, Italy) on the composition and function of the gut microbiota as well as on selected functional and metabolic parameters in children with PKU. The nutritional composition of the PS used in this study is reported in Table 1.

## 2. Materials and Methods

### 2.1. Patients’ Enrollment

Thirteen patients were enrolled in two Italian centers: the Pediatric Department of ASST Santi Paolo e Carlo in Milan and at the UOSD Metabolic Diseases of AORN Santobono-Pausilipon in Naples. Inclusion criteria were: PKU diagnosis by newborn screening, age ≥ 6 years, and compliance to diet with annual mean Phe levels within the range (i.e., 120–360 µmol/L) in childhood (<12 years) and 120–600 mmol/L in adolescence and adult age (>12 years), as recommended by the European PKU guidelines [1,17]. Phe values were calculated as the average among measurements in the 6 months before the intervention and the value collected at enrolment. Patients with PKU who were following a diet with glycomacropeptide (GMP)-based protein substitutes, or who were taking prebiotics or probiotics, undergoing antibiotic treatment, or suffering from chronic or acute intestinal diseases within the 3 months prior to study initiation were excluded.

### 2.2. Collection of Clinical Data and Biochemical Status and Nutritional Assessment

Intervention with slow-release PS consisted of replacing at least 50% of usual protein intake from the Phe-free PS as slow-release formulation. The nutritional intervention lasted 4 months. At baseline (T0) and after 4 months (T1), the following analyses were performed: clinical examination (including physical examination, Tanner score evaluation and Bristol Stool Chart); hematological and biochemical evaluation including complete blood count and metabolic and nutritional parameters according to local routines for PKU. Analyses included plasma Phe/Tyr, fasting glucose, fasting insulin, LDL, HDL, total cholesterol, triglycerides, albumin, and transthyretin. Weight, height, and body composition data were collected by metabolic dietitians, who performed body composition analysis (bio-impedentiometry) and collection of dietary habits. Children’s food intake was recorded using prospective 3-day weighed food records; quantification and analysis of energy intake and nutrient composition were performed with a dedicated PC software program (MètaDieta, 2013, San Benedetto del Tronto, Italy).

### 2.3. Fatty Acids Quantification and Gut Microbiota Sequencing and Analysis

Fecal fatty acid (FA) quantification was performed by gas chromatography–mass spectrometry (GC-MS). Feces were weighed (200 mg), suspended in double-distilled water (1 mL), and mixed. An aliquot corresponding to 60 mg of feces (300 µL) was acidified with 200 µL of pure orthophosphoric acid (85%), diluted with water to 700 µL, and extracted with diethyl ether-heptane (500 µL, 1:1 *v*/*v*). The organic layer was collected for the analysis with an 8860 GC system (Agilent Technologies, Santa Clara, CA, USA) coupled to MSD 5977C (Agilent Technologies). The GC was equipped with a DB-WAX Ultra Inert column (Agilent Technologies). GC–MS conditions were as follows: injection volume 1 µL; split ratio 10:1; helium flow rate 1.2 mL/min. The injection, transfer line, quad and ion source temperatures were set at 250 °C, 250 °C, 150 °C and 230 °C. The column temperature program started at 120 °C (held for 2 min), increased to 140 °C at a rate of 5 °C/min (held for 3 min), and then ramped to 250 °C at a rate of 20 °C/min, maintaining this temperature for 24 min. Quantification of the FAs was obtained through calibration curves of pure acids in concentrations between 0.3125 mM and 5 mM. Analyte peak areas were normalized to the responses of 2-ethylbutyric acid and heptadecanoic acid, used as internal standards. FA concentrations were expressed as nmol/mg feces. Gut microbiota characterization was performed at the time-points T0 (baseline) and T1, thus collecting two samples from each patient. Stools were kept at −80 °C until use. Fecal DNA extraction was performed using the QIAamp PowerFecal Pro DNA kit (Qiagen, Hilden, Germany) according to the manufacturer’s instructions and stored until use at −20 °C. The sequencing of the V3–V4 hypervariable regions of the bacterial 16S rRNA gene was performed in service by Macrogen (Seoul, Republic of Korea), according to the Illumina 16S Metagenomic Sequencing (Illumina, San Diego, CA, USA). Paired-end 16S rRNA sequencing data underwent denoising and quality filtering via the DADA2 pipeline (v1.18.0) to generate amplicon sequence variants (ASVs). All downstream community analyses, including α-diversity metrics and β-diversity distance calculations, were then carried out in R using the phyloseq package (v1.34.0) with custom adaptations [18]. To assess intra-sample diversity (α-diversity), we calculated multiple indices, including Chao1 richness, Shannon diversity, observed ASV count (i.e., Observed Species), and Faith’s phylogenetic diversity (i.e., PD whole tree). Inter-sample differences (β-diversity) were explored using both weighted and unweighted UniFrac distances, visualized through principal coordinate analysis (PCoA) [19]. UniFrac is a phylogeny-based distance metric that quantifies differences between microbial communities by measuring the fraction of unique and shared branch lengths in a phylogenetic tree. Weighted UniFrac accounts for the relative abundance of taxa, whereas unweighted UniFrac considers only their presence or absence. For the taxonomic classification of ASVs, we employed the Genome Taxonomy Database (GTDB 16S rRNA release r207) [20] using the RDP classifier [21].

### 2.4. Statistical Analysis

Continuous variables are expressed as mean ± standard deviation (SD), and relative abundances are reported as percentages. Paired comparisons between pre- and post-intervention measurements were carried out using the Wilcoxon signed-rank test. Two-sided *p*-values < 0.05 were considered as statistically significant.

### 2.5. Ethical Approval

The study was conducted according to the guidelines of the Declaration of Helsinki and approved by the Ethics Committee (Comitato Etico Milano Area 1, Protocol Number 2015/ST/135, 2020/EM/192).

## 3. Results

### 3.1. Cohort and Intervention Description

Thirteen patients with PKU were enrolled in this study, with a slight prevalence of males (7/13 patients). The median age at baseline was 17 years (IQR: 14–21). Nine patients had classic PKU (c-PKU), established on the basis of the pre-treatment value, using >1200 µmol/L as the cut-off. The aim of the dietary intervention was to replace at least 50% of the protein equivalent (P.Eq.) intake from Phe-free PS (e.g., amino acid mixtures) with P.Eq. deriving from the slow-release PS. On average, 78% of the P.Eq. was consumed from the slow release PS. All patients consumed at least 50%, as prescribed, and 5 of them even reached 100% within 4 months.

### 3.2. Biochemical Data Evaluation

Plasma Phe, Tyr, and their ratio (Phe/Tyr) were measured at baseline (T0) and after intervention (T1) in 13 subjects. Mean Phe levels slightly increased from 368 ± 174 µmol/L at T0 to 384 ± 172 µmol/L at T1 (*p* = 0.4846). Tyr levels showed a minor increase from 40 ± 8.6 to 45 ± 15 µmol/L (*p* = 0.3453). The Phe/Tyr ratio decreased slightly from 9.5 ± 4.7 to 9.3 ± 4.7 (*p* = 0.8067). However, due to high inter-subject variability, none of these changes have reached statistical significance.

Fasting plasma glucose showed a slight decrease from baseline to post-intervention (T0: 84.2 ± 6.2 mg/dL; T1: 79.7 ± 7.4 mg/dL; *p* = 0.0802). Similarly, fasting insulin levels decreased significantly (T0: 9.5 ± 4.9 µU/mL; T1: 7.2 ± 3.5 µU/mL; *p* = 0.0185). Insulin resistance, assessed by HOMA-IR, significantly decreased after the intervention, from 2.0 ± 1.1 at T0 to 1.4 ± 0.7 at T1 (*p* = 0.0099). Conversely, beta-cell function measured by HOMA-B showed a non-significant increase from 152.9 ± 69.8 to 220.3 ± 193.9. Fasting insulin sensitivity, evaluated by QUICKI, significantly improved from 0.6 ± 0.1 to 0.7 ± 0.1 (*p* = 0.0404).

### 3.3. Nutritional Composition and Dietary Intake

Anthropometric parameters remained stable between T0 and T1. Mean body weight was 56.35 kg at baseline and 56.75 kg after 4 months, with BMI values of 22.2 and 22.1 kg/m^2^, respectively. Body fat mass did not change significantly (21.6 ± 8.4% at T0 vs. 20.2 ± 10.1% at T1), while lean mass remained comparable (78.6 ± 8.6% vs. 79.7 ± 10.0%). Overall, no impact of the product was observed on body composition.

Dietary intakes did not differ significantly between T0 and T1. Total energy intake decreased slightly from 1739.79 ± 351.61 to 1694.58 ± 334.06 kcal/day (*p* = 0.6750), while protein, fat, carbohydrate, and fiber intake remained comparable across time points. In particular, both total protein intake and protein equivalent intake from PS remained stable, as expected according to the prescribed diet. The detailed values for each nutrient are presented in Table 2.

### 3.4. Gut Microbiota Analysis

**Figure 1:**
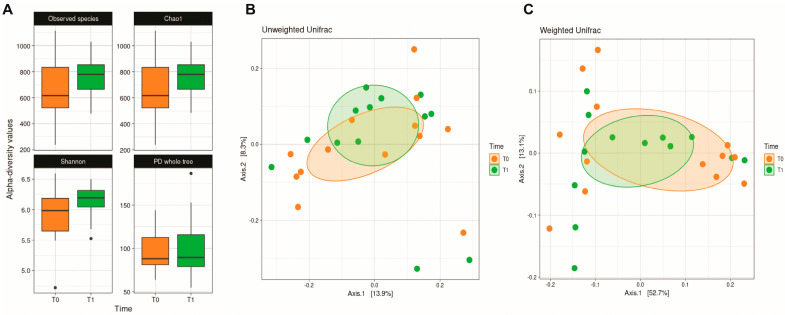
Boxplots of α-diversity metrics at baseline (T0, orange, n = 13) and after intervention (T1, green, n = 13). No statistically significant differences were observed in the Observed Species (*p* = 0.420), Chao1 (*p* = 0.420), Shannon (*p* = 0.223), and PD Whole Tree (*p* = 0.814) indices (**A**). Principal coordinate analysis (PCoA) plots illustrate β-diversity at T0 (orange) and T1 (green), using the unweighted UniFrac (**B**) and weighted UniFrac (**C**) metrics. No significant differences were observed between the two time points (unweighted UniFrac *p* = 0.9825; weighted UniFrac *p* = 0.716).

Alpha diversity analysis (Figure 1) did not reveal statistically significant differences driven by the dietary intervention. However, a modest improvement in microbial richness and evenness was observed, with both Observed Species (*p* = 0.420), Shannon (*p* = 0.223) and Chao1 (*p* = 0.420) indices showing higher values post-intervention compared to baseline. Similarly, β-diversity analysis did not reveal significant differences in microbiota composition between the time points, as the groups largely overlap.

**Figure 2:**
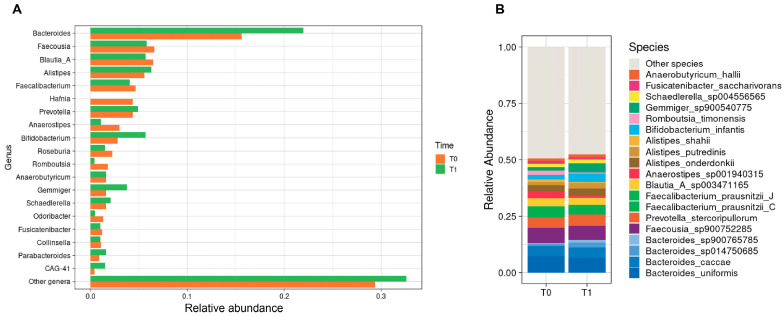
Gut microbiota composition in PKU patients (n = 13) before and after slow-release L-AAs supplementation. (**A**) Relative abundance of dominant bacterial genera. (**B**) Relative abundance of dominant bacterial species.

Figure 2 illustrates the relative abundance of the most abundant bacterial genera in the gut microbiota of PKU patients before and after the introduction of the slow-release L-AAs supplement. Overall, no statistically significant compositional shifts were observed. *Bacteroides* increased from 0.16 to 0.22 (*p* = 0.340), paralleled by a modest increase in *Bifidobacterium* from 0.03 to 0.06 (*p* = 0.367) and *Gemmiger* from 0.02 to 0.04 (*p* = 0.784). By contrast, *Hafnia* was entirely depleted post-intervention (from 0.05 to 0; *p* = 1.000). Moreover, *Anaerostipes* declined from 0.03 to 0.01 (*p* = 0.505) and *Romboutsia* showed a downward trend from 0.01 to 0.004 (*p* = 0.055).

At the species level, similar patterns emerged. *Bifidobacterium infantis* increased from 0.02 to 0.04 (*p* = 0.456) and *Gemmiger* spp. doubled from 0.02 to 0.04 (*p* = 0.784). By contrast, *Romboutsia timonensis* declined from 0.02 to 0.004 (*p* = 0.055), paralleled by a modest drop in *Anaerostipes* spp. from 0.03 to 0.01 (*p* = 0.505).

**Figure 3:**
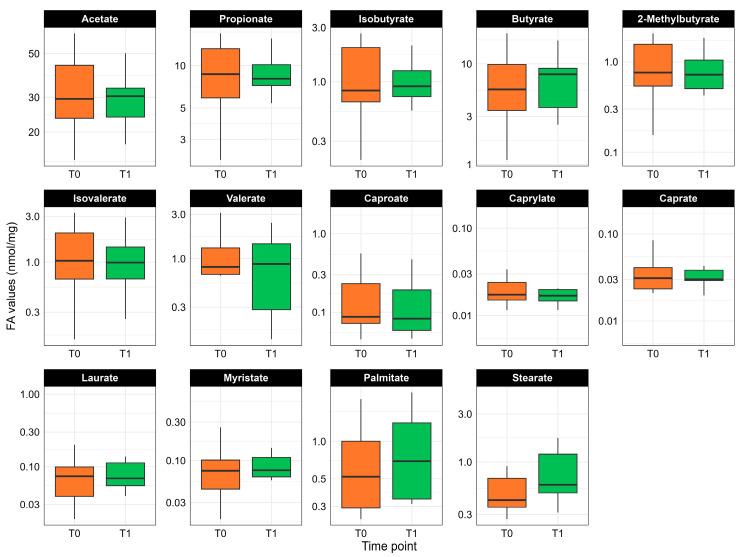
Paired comparison of fecal fatty acid concentrations (nmol/mg) in PKU patients (n = 12) before (T0, orange) and after (T1, green) slow-release L-AAs supplementation. Each panel is a different fatty acid (names shown in-graph).

Paired measurements of fecal fatty acid concentrations are displayed in Figure 3. One sample was excluded from fatty acid profiling due to insufficient material, so all analyses were carried out on the remaining 12 PKU patients. Although none of the comparisons reached statistical significance, some fatty acids trended upward following slow-release L-AA supplementation. In particular, median levels of butyrate (*p* = 0.724), palmitate (*p* = 0.289), and stearate (*p* = 0.255) showed modest increases post-diet. By contrast, the branched-chain fatty acids isobutyrate (*p* = 0.666), 2-methylbutyrate (*p* = 0.610), and isovalerate (*p* = 0.666) remained essentially unchanged following the slow-release L-AAs supplementation.

## 4. Discussion

This work represents the first study to evaluate the effect of a slow-release Phe-free PS on gut microbiota composition in patients with PKU. In addition to microbiota profiling, the investigation also included the evaluation of nutritional status and biochemical parameters to provide a more comprehensive assessment of the intervention. From a nutritional perspective, no changes were observed in body weight or body composition. This stability can be attributed to the maintenance of comparable macronutrient intake, while total energy intake showed a slight but non-significant decrease. The dietary protocol was specifically designed to minimize confounding factors and ensure that observed outcomes could be more directly ascribed to the intervention. Biochemical analyses related to amino acid metabolism revealed no statistically significant alterations, although plasma Phe concentrations showed a modest increase trend, whereas tyrosine concentrations declined. In contrast, indices of glucose–insulin homeostasis demonstrated more important modifications. Plasma glucose and insulin levels decreased, accompanied by a significant reduction in HOMA-IR, indicating better insulin sensitivity and consequently improved metabolic control. Although HOMA-β also decreased, this change was not statistically significant. Of particular note, insulin sensitivity, as reflected by the QUICKI index, was significantly increased, suggesting a beneficial metabolic adaptation associated with the intervention.

Previous studies have investigated the effects of the same slow-release PS in 5 patients PKU, primarily focusing on plasma amino acid concentrations [22]. Their results indicate that the use of slow-release PS does not significantly alter Phe levels compared with standard amino acids formulations but is consistently associated with higher plasma Tyr concentrations. This improvement in Tyr availability has been attributed to the continuous absorption profile of slow-release technologies, which more closely mimics the kinetics of natural protein digestion. In contrast, our study did not observe a significant improvement in Tyr concentrations. A modest reduction was noted, together with a slight upward trend in Phe. These divergent findings may reflect differences in study design, or duration of follow-up. In the cited study [22], patients fully replaced their protein sources from free amino acids to the same slow-release PS and were followed for 6 months, whereas in our study amino acids were only partially substituted and for 4 months. Moreover, the protein sources previously consumed by patients in the cited study had a lower average tyrosine content, while this information is not available for our cohort.

Preclinical studies have also investigated the metabolic impact of prolonged-release amino acid formulations [23]. In animal models, acute administration of slow-release amino acids was associated with lower blood BUN, resembling the metabolic profile of intact casein, and with a more favorable nitrogen balance compared with free amino acids. Long-term supplementation resulted in reduced expression of muscle degradation markers, increased grip strength, and improved glucose tolerance. Importantly, these findings suggest that delayed amino acid absorption may reduce amino acid oxidation and protein catabolism, while exerting beneficial influences on glucose metabolism and muscle function. Although extrapolation from animal to human models requires caution, these results support the hypothesis that slow-release PS may favorably modulate insulin sensitivity and glycemic control. This is consistent with our study, in which significant improvements were observed in HOMA-IR and QUICKI index, indicating enhanced insulin sensitivity. These results highlight a potential role of slow-release formulations not only in sustaining amino acid homeostasis, but also in mitigating the risk of metabolic complications, such as insulin resistance, which is of particular concern in PKU patients due to the high carbohydrate content of their diet [12,24].

Finally, recent case series [25] have reported that slow-release PS were well tolerated in pregnant women with PKU, allowing good metabolic control and favorable pregnancy outcomes. Although no detailed nutritional or biochemical data were provided, these findings suggest that slow-release PS may also be a good clinical option in the challenging context of maternal PKU. Future studies should further investigate their potential to improve both maternal tolerance and fetal development.

Amino acid composition, release kinetics and intestinal absorption directly influence substrate availability to the colonic microbiota. Slow-release formulations are designed to slow small-intestinal uptake and/or change the temporal profile of luminal amino acids reaching the distal gut, thereby increasing the fraction of amino-nitrogen and carbon that becomes available to commensal microorganisms. In theory, this can favor taxa capable of utilizing free amino acids or cross-feeding on their fermentation products, thereby reshaping or restoring ecological niches [26,27,28].

Our intervention with a slow-release amino acid supplement produced only modest shifts in the gut microbiota of PKU patients. Alpha and β-diversity metrics were stable after supplementation, a result that echoes other clinical reports showing only minor effects of low-protein or formula-based dietary interventions on overall richness and diversity but noted consistent family- and species-level changes [29]. These data also agree with previous findings reporting that PKU dietary interventions often leave diversity indices unchanged [15,30].

Nonetheless, several taxa displayed changes that, although not statistically significant, may be biologically meaningful. *Bifidobacterium* showed an upward trend following supplementation. This is in line with prior evidence that PKU formulas containing GMP and carbohydrates can act as prebiotics for *Bifidobacteria* [31]. Human and preclinical data suggest GMP is microbiologically safe and may favor specific saccharolytic taxa in PKU [30,31]. Mechanistically, these bacteria ferment oligosaccharides to produce acetate and lactate, which then act as substrates for butyrate producers via cross-feeding. In this way, *Bifidobacterium* indirectly contributes to colonic butyrate pools, which are central to gut and immune homeostasis. Beyond cross-feeding, *Bifidobacteria* (including *B. infantis*) also exert direct host benefits: acetate has been shown to strengthen the intestinal barrier and to stimulate mucus secretion, while immune modulation through IL-10 induction and expansion of regulatory T cells further highlights its protective potential [32,33,34]. Consistent with this, we also observed an upward trend in *Gemmiger*, a known butyrate-producing taxon. Several studies have reported positive correlations between *Gemmiger* abundance and fecal butyrate [35,36]. The co-occurrence of modestly increased *Bifidobacterium* (producer of acetate/lactate) and expansion of *Gemmiger* supports a saccharolytic cross-feeding model in which bifidobacterial fermentation supplies substrates that downstream butyrogenic taxa convert to butyrate [37].

*Bacteroides* also increased modestly. This expansion is a well-described response to greater luminal availability of peptides and amino acids, as shown in short-term dietary interventions [38,39]. Functionally, many *Bacteroides* species are metabolically versatile and can metabolize both complex polysaccharides and protein-derived substrates; therefore, their increase can reflect a selective advantage in environments with greater peptide/amino-acid availability rather than an intrinsically pathogenic change [40,41]. Importantly, in our study branched-chain SCFAs markers of proteolytic fermentation did not increase, suggesting that this *Bacteroides* enrichment did not translate into deleterious proteolysis [42].

By contrast, *Romboutsia* showed a downward trend. Several population and case–control studies report higher *Romboutsia* abundance in healthy comparators and depletion in disease states, prompting the suggestion that *Romboutsia* may in some contexts track mucosal or metabolic integrity [43,44]. However, Romboutsia is diet-responsive in intervention studies and its relative fitness appears sensitive to changes in dietary substrate, and competitive cross-feeding dynamics; thus, the observed decline may simply reflect niche displacement by taxa favored under the altered intraluminal conditions created by the slow-release amino-acid supplement [45,46].

Consistent with the microbiota changes, we saw trends toward higher fecal butyrate and slight increases in saturated long-chain fatty acids. Although none reached statistical significance, median butyrate rose post-supplementation. This is noteworthy because conventional PKU diets are often deficient in butyrate-producers and butyrate itself [15,31]. Butyrate is a crucial SCFA, as it is the preferred energy source for colonic epithelial cells and strengthens tight junctions, thereby enhancing barrier function and also has anti-inflammatory effects (e.g., via regulatory T-cell induction) [47,48]. Our finding of increased butyrate is consistent with the expansion of butyrate-producing genera. The lack of branched SCFAs (isobutyrate, 2-methylbutyrate, isovalerate) suggests that global proteolytic fermentation in the colon was not markedly altered by the slow-release amino acid formula. Interestingly, fecal palmitate and stearate levels showed a modest upward trend after treatment. Saturated long-chain fatty acids in stools may reflect dietary fat intake, altered lipid absorption or microbial lipase activity. High intake of palmitate has been linked to impaired gut barrier and inflammation [49], whereas stearic acid has been correlated with the promotion of rat colonic muscle contraction and increase stool frequency [50]. It is possible that an improved microbial balance allowed slightly more fat to escape into the stool, or that altered transit times affected the lipid absorption. The clinical significance of these changes in fatty acids remains unclear and requires further study.

This study has several limitations. First, the small cohort size limits the generalizability of the findings. Although no statistically significant differences were detected, we observed interesting trends in several microbial taxa and metabolites that may still indicate biological effects. Larger studies are needed to confirm these observations and to determine whether they represent stable or transient adaptations. Second, the duration of the intervention was relatively short. Gut microbial communities, particularly in individuals who have followed a PKU-specific diet for many years, may require longer exposure to dietary modifications before measurable compositional shifts occur. Finally, the participants’ age may have influenced the microbiota’s responsiveness. During early life, the gut microbiome is still developing and exhibits greater plasticity to dietary and environmental factors, whereas in older adolescents and adults, the ecosystem tends to be more stable and less prone to rapid change. Given the age of our cohort, the limited microbial shifts observed may partly reflect this reduced adaptability.

## 5. Conclusions

In summary, this study provides the first integrated evaluation of the metabolic, nutritional, and microbiota effects of a slow-release Phe-free PS in patients with PKU. Despite the small cohort and short intervention period, the results indicate that this formulation is safe, well-tolerated, and confirms to be capable of improving selected metabolic parameters, most notably insulin sensitivity, without negatively affecting amino acid homeostasis or nutritional status.

At the gut microbiota level, overall diversity remained stable, but coherent trends suggested subtle functional adaptations, including increases in saccharolytic and butyrate-associated taxa such as *Bifidobacterium* and *Gemmiger*, accompanied by a modest rise in fecal butyrate. While the relatively older age of participants may have limited the extent of microbial remodeling, this also highlights an important translational perspective: introducing similar nutritional strategies earlier in life could help shape the developing microbiota toward a more balanced and metabolically favorable configuration, potentially reinforcing long-term gut and metabolic health in PKU.

Taken together, these findings support the potential of slow-release amino acid formulations not only to optimize amino acid absorption and metabolic control, but also to contribute to microbiota homeostasis. Larger, age-stratified, and longer-term studies are warranted to confirm these effects and to explore their preventive potential when implemented from early developmental stages.

## Figures and Tables

**Table 1:** Nutritional composition of the slow-release L-AAs PS used in the PREMP Study. This product can be used from 3 years of age.

Nutritional Values	Mean Values per 100 g
Energy (Kj)	1678
Energy (Kcal)	396
Total Fats (g)	3.6
Saturated Fats (g)	3.59
Carbohydrates (g)	13
Sugars (g)	0
Total Fibre (g)	3.7
Total Equivalent Proteins (g)	70.7
L-Alanine (g)	3.07
L-Arginine (g)	4.9
L-Aspartic-Acid (g)	7.76
L-Cystine (g)	2.01
Glycine (g)	7.68
L-Glutamine (g)	6.02
L-Histidine (g)	3.07
L-Isoleucine (g)	5.31
L-Leucine (g)	8.27
L-Lysine (g)	5.5
L-Methionine (g)	1.42
L-Phenylalanine (g)	0
L-Proline (g)	5.54
L-Serine (g)	3.42
L-Threonine (g)	5.31
L-Tryptophan (g)	1.65
L-Tyrosine (g)	7.78
L-Valine (g)	6.13
L-Carnitine (g)	0.08
L-Taurine (g)	0.12
Salt (g)	1

**Table 2:** Dietary intake at T0 and T1. Amounts are reported as mean and standard deviations (SD). *p*-values <0.05 were considered significant.

Dietary Intake	T0 (Mean ± SD)	T1 (Mean ± SD)	*p*-Value
Energy (kcal)	1739.79 ± 351.61	1694.58 ± 334.06	0.6750
Protein (g)	56.84 ± 18.62	62.63 ± 14.88	0.2635
Protein (%)	14.26 ± 5.64	15.02 ± 3.65	0.1822
Natural protein (g/kg/day)	0.38 ± 0.25	0.38 ± 0.23	1.0000
Protein equivalent from PS (g/kg/day)	0.78 ± 0.33	0.89 ± 0.27	0.0692
Phe (mg/day)	872.48 ± 658.66	808.00 ± 511.31	0.6247
Fat (g)	63.05 ± 12.41	58.95 ± 10.98	0.1261
Fat (%)	38.06 ± 9.86	36.45 ± 10.26	0.1842
Carbohydrates (g)	236.88 ± 57.91	236.16 ± 66.01	0.8888
Carbohydrates (%)	47.00 ± 10.50	47.74 ± 11.37	0.8339
Fiber (g)	15.86 ± 7.11	16.58 ± 7.88	0.8613

## Data Availability

The raw data supporting the conclusions of this article will be made available by the authors on request.
